# The complete chloroplast genome of *Lilium amoenum* (Liliopsida: Liliaceae) from Yunnan, China

**DOI:** 10.1080/23802359.2021.1885318

**Published:** 2021-03-15

**Authors:** Weiwei Bai, Jingzhi Wu, Shuilian He, Zhijia Gu, Hui Jiang, Hongzhi Wu

**Affiliations:** Yunnan Agricultural University, Kunming, China

**Keywords:** Chloroplast genome, *Lilium amoenum*, phylogenetic analysis

## Abstract

*Lilium amoenum* E. H. Wilson ex Sealy is classified in Liliaceae, and it is an important ornamental plant with wonderful rose-red color and pleasant rose fragrance. In this study, we sequenced the complete chloroplast genome of *L. amoenum* by Illumina Hiseq X Ten and PacBio RS technologies. The genome size of *L. amoenum* is 152,280 bp, and displays a typical quadripartite structure: one large single-copy (LSC, 81,977 bp), one small single-copy (SSC, 17,539 bp), and a pair of inverted repeat regions (IRs, 26,382 bp). The overall GC content was 37.0%. The complete genome contained 131 genes, including 85 protein-coding genes, 38 tRNA genes, and 8 rRNA genes. Phylogenetic analysis showed that *L. amoenum* is closely related to *L. taliense* and *L. bakerianum*. The present study could afford crucial genetic information for further researches on the genus and related genera.

*Lilium* is an important ornamental flower used as a cut flower, pot plant and garden plant. However, there are some obvious problems in the existing cultivars, including variation in fragrance and plant size (Huang 1983). For *Lilium*, orange and white flowers are the common, but the rose-red colored flowers are rare (Huang 1983). *Lilium amoenum*, originally from the Yunnan province of China and distributed at altitudes 1900–2500 m, is a unique species with dwarf plant, wonderful rose-red color and pleasant rose fragrance (De Jong 1974). *Lilium amoenum* is classified in the section Lophophorum of the Liliaceae (De Jong 1974), and is an attractive resource for germplasm innovation and breeding varieties (Huang et al. 1990; Pan et al. 2018). Due to its confined distribution, human collection and serious ecological environment damage, the natural distribution of *L. amoenum* in the wild is becoming scarce, and may be soon endangered (Wu et al. 2010). Therefore, it is extremely urgent to develop the genetic resources to protect and use this germplasm. In the present study, we determined the chloroplast genome sequence of *L. amoenum*, and discussed the genetic relationship among various species in the Liliaceae.

The fresh leaves were collected from the *L. amoenum* planted in the germplasm nursery of Yunnan Agricultural University (104°30′30“E, 23°12′50“N). The voucher specimen was deposited at the Herbarium of Yunnan Agricultural University (No. 2020WHZ004, Shuilian He, heshuilian2006@163.com). Total genomic DNA was isolated from fresh leaves using a DNeasy Plant Mini Kit (QIAGEN, Valencia, California, USA) according to the manufacturer’s instructions. The obtained DNA was constructed into average 350 bp paired-end (PE) library by Illumina Hiseq platform (Illumina, San Diego, CA, USA) and sequenced using an Illumina NovaSeq platform.

The output was a 4 Gb raw data of 150 bp paired-end reads. Prior to chloroplast de novo assembly, low-quality reads were filtered out and resultant clean reads were assembled using the default settings in SPAdes (Bankevich et al. 2012). The resulting clean reads were assembled using GetOrganelle pipeline (https://github.com/Kinggerm/GetOrganelle). The genome was automatically annotated using the CpGAVAS pipeline (Liu et al. 2012) and start/stop codons and intron/exon boundaries were adjusted in Geneious 8.1 (Kearse et al. 2012), and inspected by comparison against the *L. taliense* complete chloroplast genome (GenBank accession number: KY009938).

The results of whole genome sequencing showed that the size of chloroplast genome of *L. amoenum* (Genbank accession number: MT880912) was 152,280 bp with a typical tetragonal structure: one large single-copy (LSC, 81,977 bp), one small single-copy (SSC, 17,539 bp) and two reverse repeats (IRS, 26,382 bp). The total GC content was 37.0%, the GC content of large single copy (LSC) was 34.8%, and the GC content of small single copy (SSC) was 30.6%. A total of 131 genes were detected, including 85 protein coding, 38 tRNA and 8 rRNA genes. Twenty-one gene are partially or completely duplicated, including seven PCG (rpl2; rpsl23; ycf2; ndhB; rps12; rps7; ycf1), ten tRNA (two trnI-GAU, two trnA-UGC, trnL-CAA, trnI-CAU, trnR-ACG, trnV-GAC, trnN-GUU, trnH-GUG) and all four rRNA genes (4.5S, 5S, 16S & 23S rRNA). All the rRNA genes in the genome sequence were located in the repeat region.

The phylogenetic analysis included 26 complete chloroplast genomes, 22 Lilium species and four outgroups taxa. The phylogenomic relationship was inferred by the maximum likelihood (ML) method based on the general time-reversible (GTR) + Gamma substitution model in PhyML 3.0 (Larkin et al. 2007; Guindon et al. 2010), with 1000 bootstrap replicates (Letunic and Bork 2016). Phylogenetic analysis full resolved *L. amoenum* in a clade containing *L. bakerianum* and *L. taliense* in a monophyletic Lilium. This complete cp genome provides valuable information for population genomic studies, DNA barcoding, and conservation genetics (Figure 1).

## Data Availability

The genome sequence data that support the findings of this study are openly available in GenBank of NCBI at https://www.ncbi.nlm.nih.gov/ under the accession no. MT880912. The associated BioProject, SRA, and Bio-Sample numbers are SRP287370, SRX9292592, and SRS7519269, respectively. A phylogenetic tree of the *Lilium* species based on the completed chloroplast genomes of 22 species and 4 outgroup taxa. Boostrap support values are cited at the nodes based on 1000 replicates.

## References

[CIT0001] Bankevich A, Nurk S, Antipov D, Gurevich AA, Dvorkin M, Kulikov AS, Lesin VM, Nikolenko SI, Pham S, Prjibelski AD, et al. 2012. SPAdes: a new genome assembly algorithm and its applications to single-cell sequencing. J Comput Biol. 19(5):455–477. 22506599 10.1089/cmb.2012.0021 PMC3342519

[CIT0002] De Jong PC. 1974. A new some notes on the evolution of lilies. Liliy Yearbook North Am Lily Soc. 27:23–28.

[CIT0003] Guindon S, Dufayard JF, Lefort V, Anisimova M, Hordijk W, Gascuel O. 2010. New algorithms and methods to estimate maximum-likelihood phylogenies: assessing the performance of PhyML 3.0. Syst Biol. 59(3):307–321. 20525638 10.1093/sysbio/syq010

[CIT0004] Huang JM, Zhao XY, Z GM, Ni YY. 1990. Interspecific. Hybrids by using *Lilium amoenum* as pollen parent. Acta Horticulturae Sinica. 17(2):153–154.

[CIT0005] Huang JM. 1983. Interspecific hybridization of *Lilium* was conducted with *Lilium amoenum* as parent. Chin J Nature. 4:316.

[CIT0006] Kearse M, Moir R, Wilson A, Stones-Havas S, Cheung M, Sturrock S, Buxton S, Cooper A, Markowitz S, Duran C, et al. 2012. Geneious Basic: an integrated and extendable desktop software platform for the organization and analysis of sequence data. Bioinformatics. 28(12):1647–1649. 22543367 10.1093/bioinformatics/bts199 PMC3371832

[CIT0007] Larkin MA, Blackshields G, Brown NP, Chenna R, McGettigan PA, McWilliam H, Valentin F, Wallace IM, Wilm A, Lopez R, et al. 2007. Clustal W and clustal X version 2.0. Bioinformatics. 23(21):2947–2948. 17846036 10.1093/bioinformatics/btm404

[CIT0008] Letunic I, Bork P. 2016. Interactive Tree of Life (ITOL) v3: an online tool for the display and annotation of phylogenetic and other trees. Nucleic Acids Res. 44(W1):W242–W245. 27095192 10.1093/nar/gkw290 PMC4987883

[CIT0009] Liu C, Shi L, Zhu Y, Chen H, Zhang J, Lin X, Guan X. 2012. CpGAVAS, an integrated web server for the annotation, visualization, analysis, and GenBank submission of completely sequenced chloroplast genome sequences. BMC Genom. 13:715. 10.1186/1471-2164-13-715 PMC3543216 23256920

[CIT0010] Pan YB, Wu JZ, Ou LJ, Che WG, Wu HZ. 2018. Studies on the compatibility of interspecific hybridization between wild lilies and cultivated lilies. J Yunnan Agri Univ (Nat Sci). 33(2):287–288.

[CIT0011] Wu LF, Cui GF, Zhang YP, Jia WJ, Zhao PF, Wang JH. 2010. Efficient in vitro propagation and bulb induction of *Lilium amoenum* Wilson ex Sealy. Plant Physiol J. 46(9):967–968.

